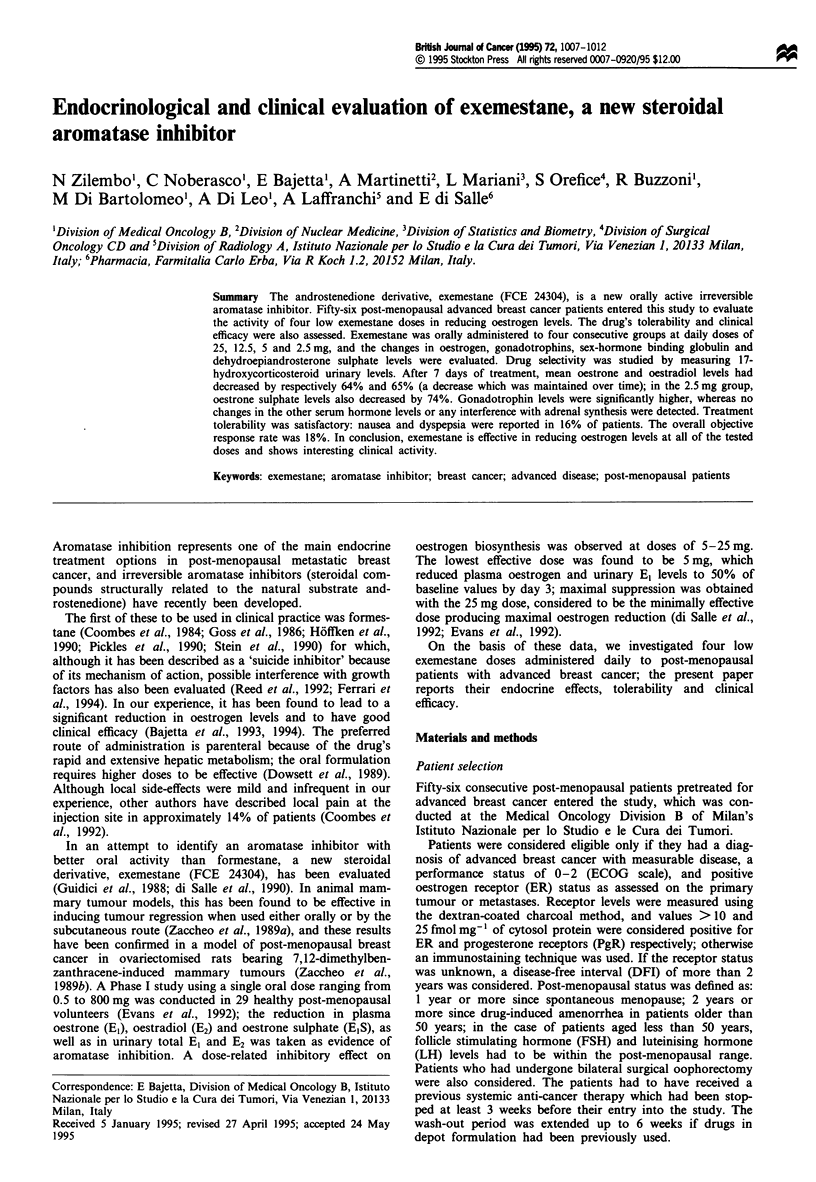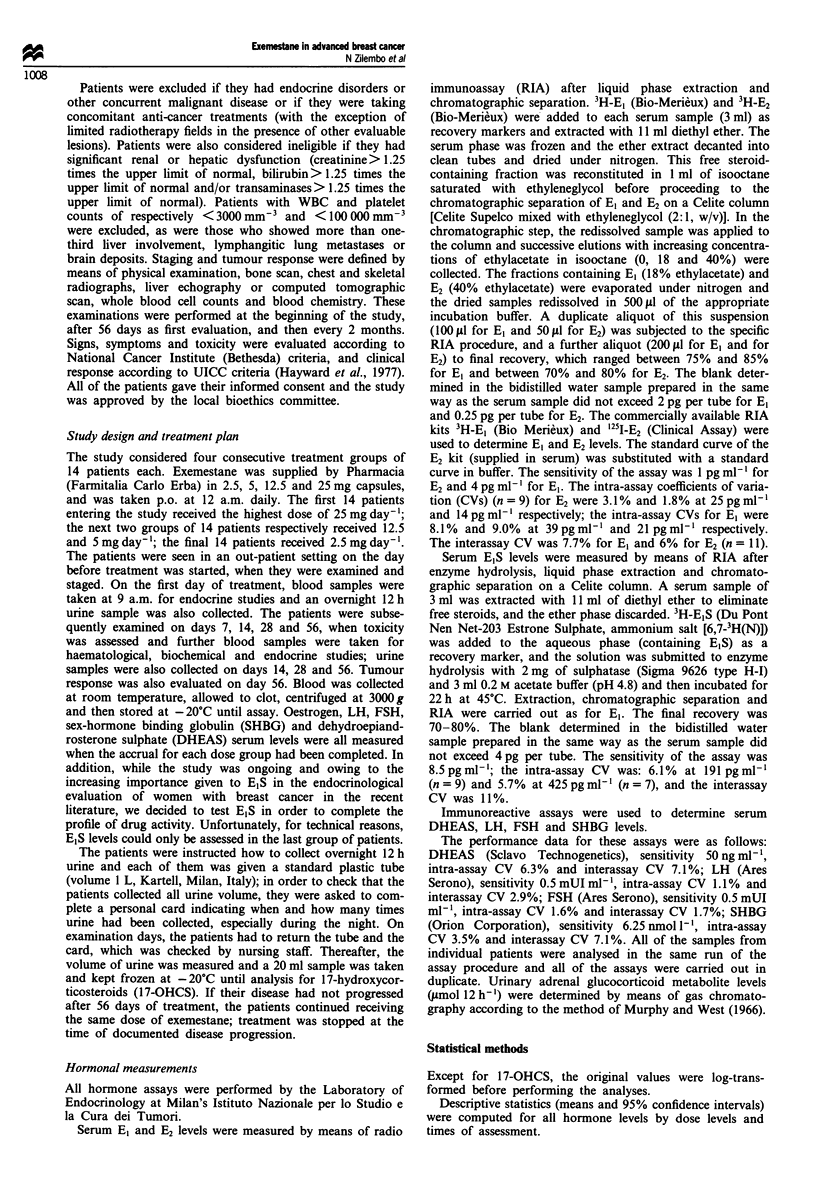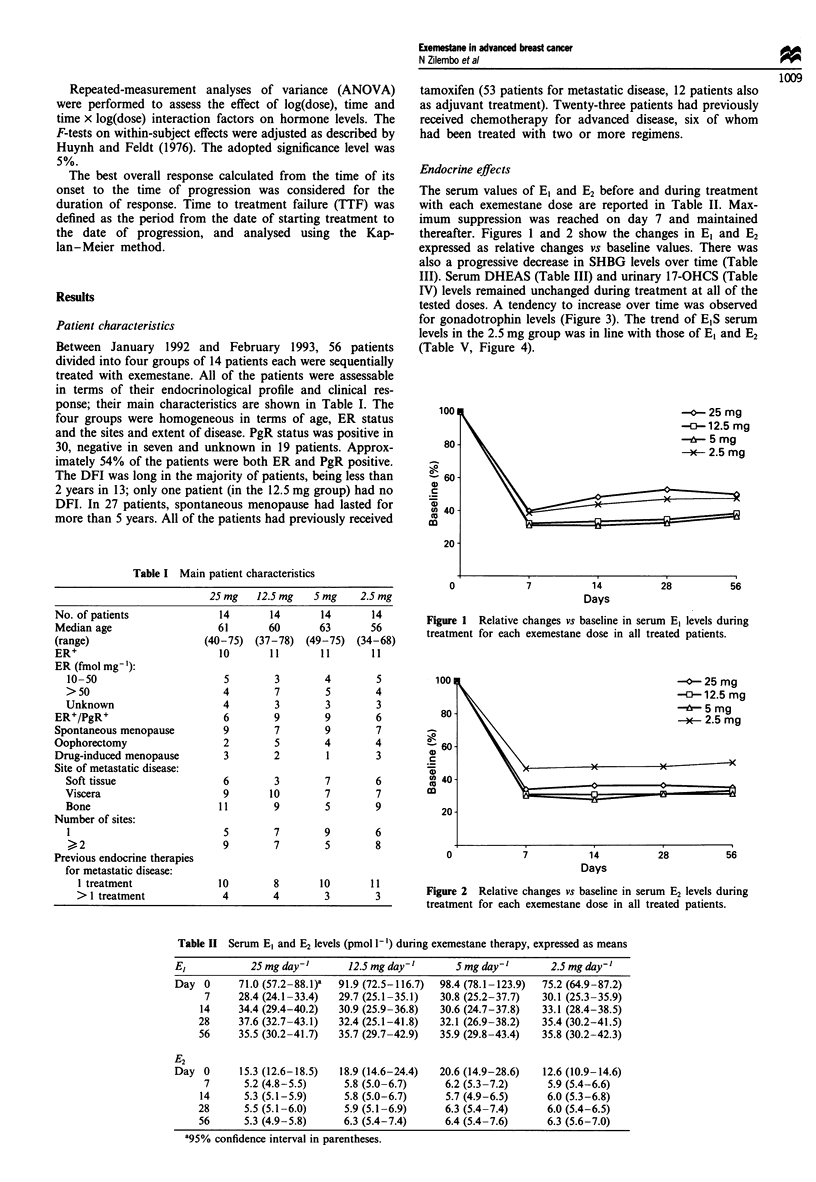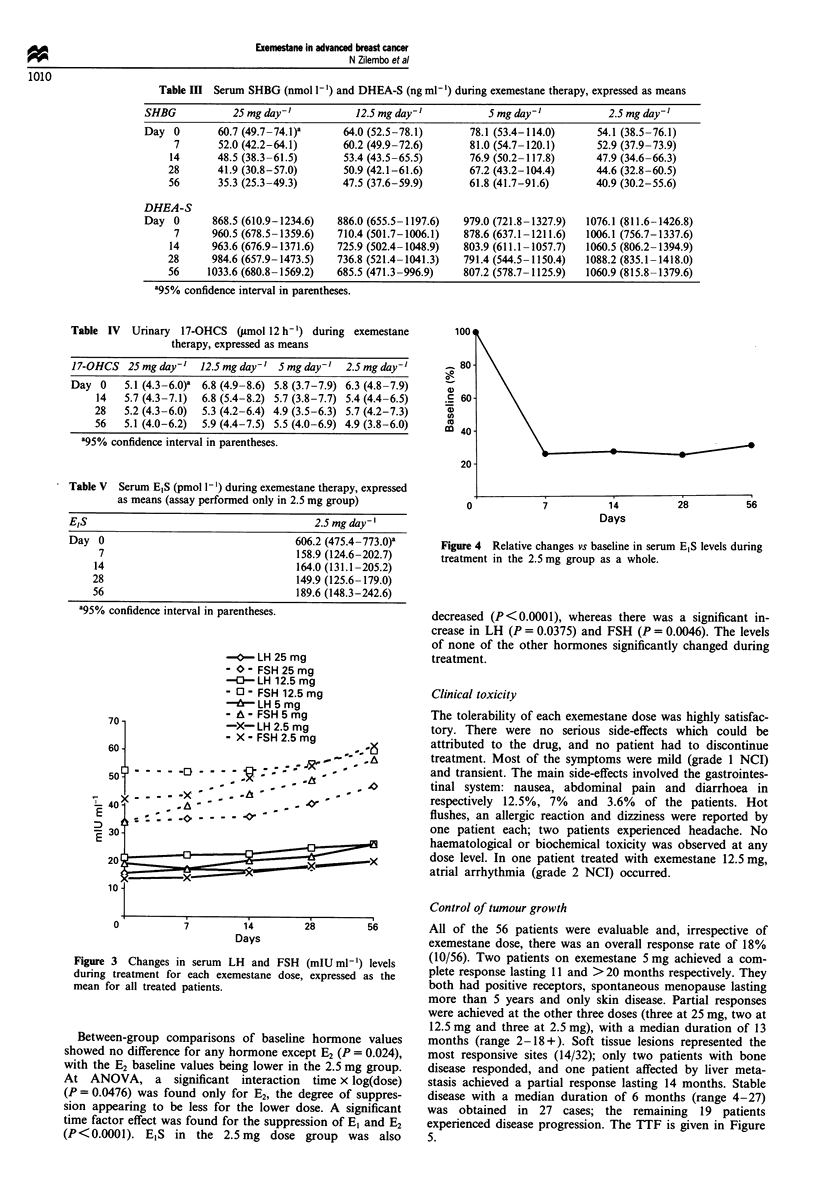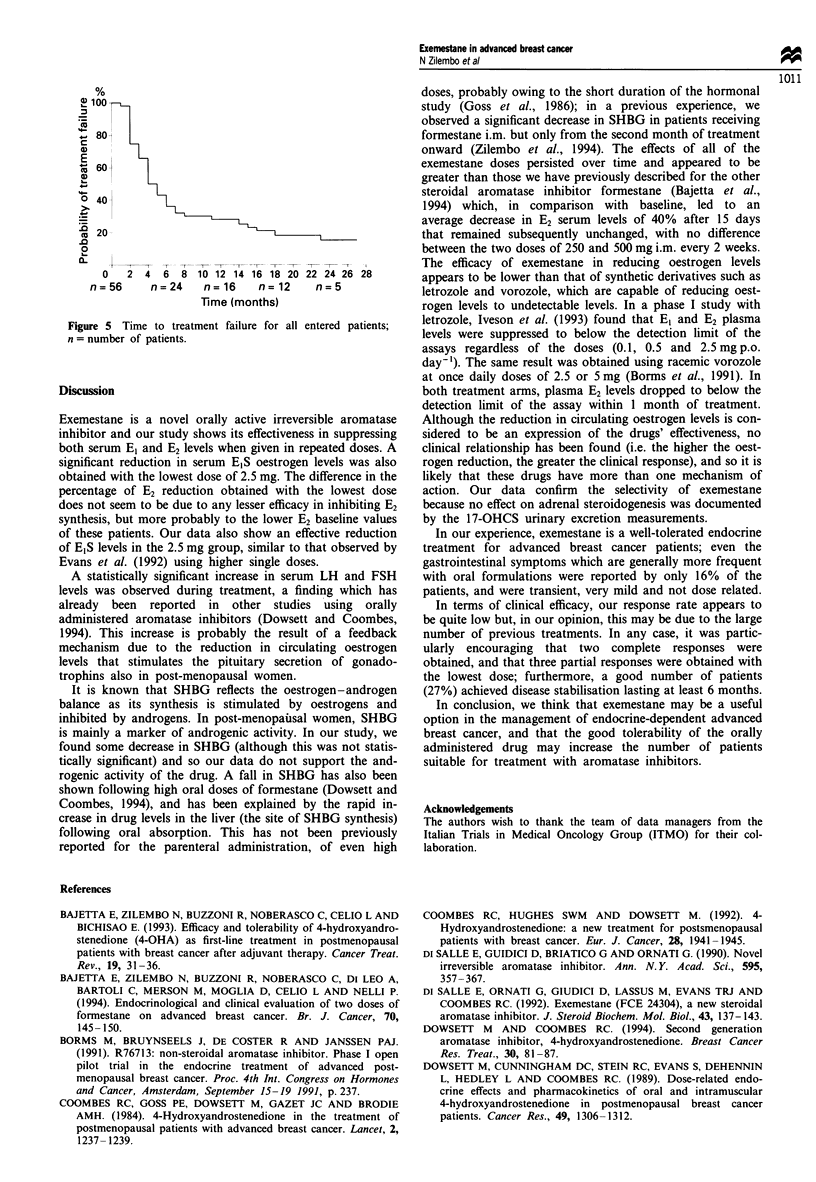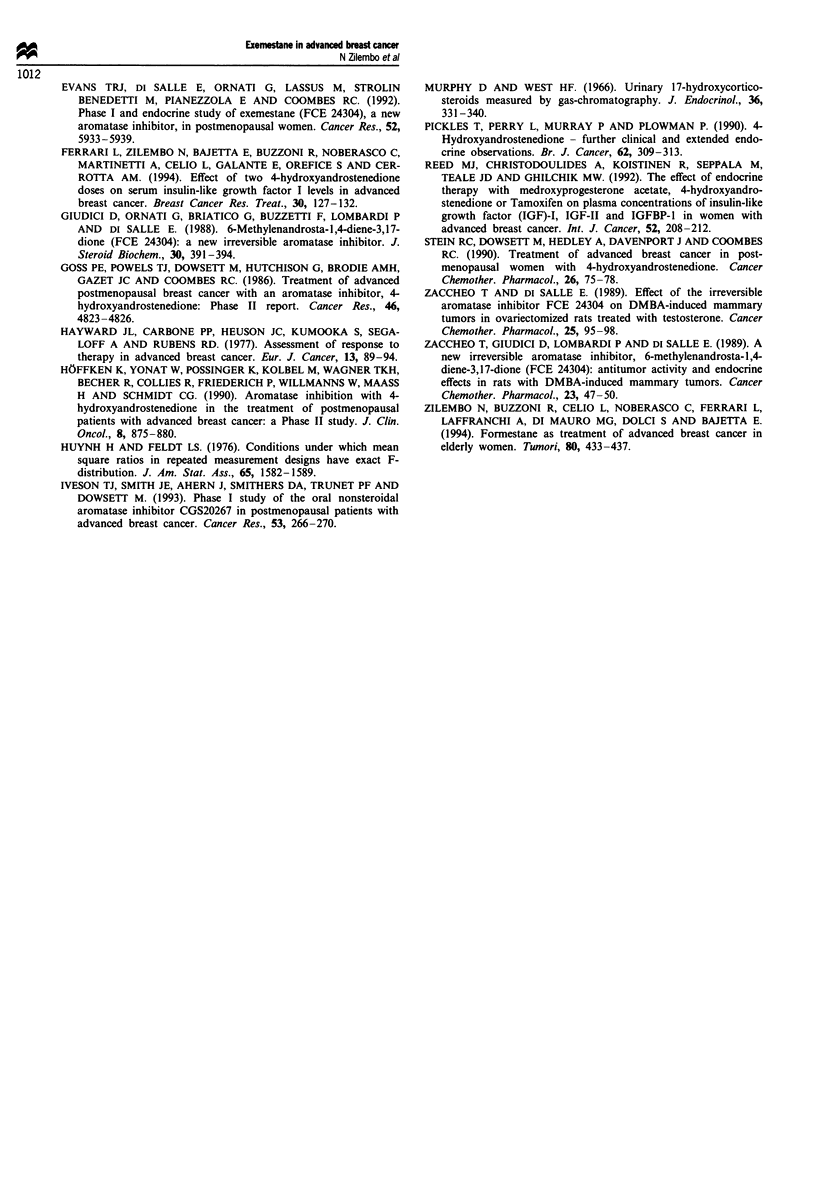# Endocrinological and clinical evaluation of exemestane, a new steroidal aromatase inhibitor.

**DOI:** 10.1038/bjc.1995.451

**Published:** 1995-10

**Authors:** N. Zilembo, C. Noberasco, E. Bajetta, A. Martinetti, L. Mariani, S. Orefice, R. Buzzoni, M. Di Bartolomeo, A. Di Leo, A. Laffranchi

**Affiliations:** Division of Medical Oncology B, Istituto Nazional per lo Studio e la Cura dei Tumori, Milan, Italy.

## Abstract

The androstenedione derivative, exemestane (FCE 24304), is a new orally active irreversible aromatase inhibitor. Fifty-six post-menopausal advanced breast cancer patients entered this study to evaluate the activity of four low exemestane doses in reducing oestrogen levels. The drug's tolerability and clinical efficacy were also assessed. Exemestane was orally administered to four consecutive groups at daily doses of 25, 12.5, 5 and 2.5 mg, and the changes in oestrogen, gonadotrophins, sex-hormone binding globulin and dehydroepiandrosterone sulphate levels were evaluated. Drug selectivity was studied by measuring 17-hydroxycorticosteroid urinary levels. After 7 days of treatment, mean oestrone and oestradiol levels had decreased by respectively 64% and 65% (a decrease which was maintained over time); in the 2.5 mg group, oestrone sulphate levels also decreased by 74%. Gonadotrophin levels were significantly higher, whereas no changes in the other serum hormone levels or any interference with adrenal synthesis were detected. Treatment tolerability was satisfactory: nausea and dyspepsia were reported in 16% of patients. The overall objective response rate was 18%. In conclusion, exemestane is effective in reducing oestrogen levels at all of the tested doses and shows interesting clinical activity.


British Journal of Cancer (1995) 72, 1007–1012
© 1995 Stockton Press All rights reserved 0007-0920/95 $12.00

### Endocrinological and clinical evaluation of exemestane, a new steroidal aromatase inhibitor

N Zilembo[1], C Noberasco[1], E Bajetta[1], A Martinetti[2], L Mariani[3], S Orefice[4], R Buzzoni[1], M Di Bartolomeo[1], A Di Leo[1], A Laffranchi[5] and E di Salle[6]

[1]*Division of Medical Oncology B,* [2]*Division of Nuclear Medicine,* [3]*Division of Statistics and Biometry,* [4]*Division of Surgical Oncology CD and* [5]*Division of Radiology A, Istituto Nazionale per lo Studio e la Cura dei Tumori, Via Venezian 1, 20133 Milan, Italy;* [6]*Pharmacia, Farmitalia Carlo Erba, Via R Koch 1.2, 20152 Milan, Italy.*

**Summary** The androstenedione derivative, exemestane (FCE 24304), is a new orally active irreversible aromatase inhibitor. Fifty-six post-menopausal advanced breast cancer patients entered this study to evaluate the activity of four low exemestane doses in reducing oestrogen levels. The drug's tolerability and clinical efficacy were also assessed. Exemestane was orally administered to four consecutive groups at daily doses of 25, 12.5, 5 and 2.5 mg, and the changes in oestrogen, gonadotrophins, sex-hormone binding globulin and dehydroepiandrosterone sulphate levels were evaluated. Drug selectivity was studied by measuring 17-hydroxycorticosteroid urinary levels. After 7 days of treatment, mean oestrone and oestradiol levels had decreased by respectively 64% and 65% (a decrease which was maintained over time); in the 2.5 mg group, oestrone sulphate levels also decreased by 74%. Gonadotrophin levels were significantly higher, whereas no changes in the other serum hormone levels or any interference with adrenal synthesis were detected. Treatment tolerability was satisfactory: nausea and dyspepsia were reported in 16% of patients. The overall objective response rate was 18%. In conclusion, exemestane is effective in reducing oestrogen levels at all of the tested doses and shows interesting clinical activity.

**Keywords**: exemestane; aromatase inhibitor; breast cancer; advanced disease; post-menopausal patients

Aromatase inhibition represents one of the main endocrine treatment options in post-menopausal metastatic breast cancer, and irreversible aromatase inhibitors (steroidal compounds structurally related to the natural substrate androstenedione) have recently been developed.

The first of these to be used in clinical practice was formestane (Coombes *et al.*, 1984; Goss *et al.*, 1986; Höffken *et al.*, 1990; Pickles *et al.*, 1990; Stein *et al.*, 1990) for which, although it has been described as a 'suicide inhibitor' because of its mechanism of action, possible interference with growth factors has also been evaluated (Reed *et al.*, 1992; Ferrari *et al.*, 1994). In our experience, it has been found to lead to a significant reduction in oestrogen levels and to have good clinical efficacy (Bajetta *et al.*, 1993, 1994). The preferred route of administration is parenteral because of the drug's rapid and extensive hepatic metabolism; the oral formulation requires higher doses to be effective (Dowsett *et al.*, 1989). Although local side-effects were mild and infrequent in our experience, other authors have described local pain at the injection site in approximately 14% of patients (Coombes *et al.*, 1992).

In an attempt to identify an aromatase inhibitor with better oral activity than formestane, a new steroidal derivative, exemestane (FCE 24304), has been evaluated (Guidici *et al.*, 1988; di Salle *et al.*, 1990). In animal mammary tumour models, this has been found to be effective in inducing tumour regression when used either orally or by the subcutaneous route (Zaccheo *et al.*, 1989*a*), and these results have been confirmed in a model of post-menopausal breast cancer in ovariectomised rats bearing 7,12-dimethylbenzanthracene-induced mammary tumours (Zaccheo *et al.*, 1989*b*). A Phase I study using a single oral dose ranging from 0.5 to 800 mg was conducted in 29 healthy post-menopausal volunteers (Evans *et al.*, 1992); the reduction in plasma oestrone ($E_1$), oestradiol ($E_2$) and oestrone sulphate ($E_1S$), as well as in urinary total $E_1$ and $E_2$ was taken as evidence of aromatase inhibition. A dose-related inhibitory effect on oestrogen biosynthesis was observed at doses of 5–25 mg. The lowest effective dose was found to be 5 mg, which reduced plasma oestrogen and urinary $E_1$ levels to 50% of baseline values by day 3; maximal suppression was obtained with the 25 mg dose, considered to be the minimally effective dose producing maximal oestrogen reduction (di Salle *et al.*, 1992; Evans *et al.*, 1992).

On the basis of these data, we investigated four low exemestane doses administered daily to post-menopausal patients with advanced breast cancer; the present paper reports their endocrine effects, tolerability and clinical efficacy.

### Materials and methods

### *Patient selection*

Fifty-six consecutive post-menopausal patients pretreated for advanced breast cancer entered the study, which was conducted at the Medical Oncology Division B of Milan's Istituto Nazionale per lo Studio e le Cura dei Tumori.

Patients were considered eligible only if they had a diagnosis of advanced breast cancer with measurable disease, a performance status of 0–2 (ECOG scale), and positive oestrogen receptor (ER) status as assessed on the primary tumour or metastases. Receptor levels were measured using the dextran-coated charcoal method, and values >10 and 25 fmol mg$^{-1}$ of cytosol protein were considered positive for ER and progesterone receptors (PgR) respectively; otherwise an immunostaining technique was used. If the receptor status was unknown, a disease-free interval (DFI) of more than 2 years was considered. Post-menopausal status was defined as: 1 year or more since spontaneous menopause; 2 years or more since drug-induced amenorrhea in patients older than 50 years; in the case of patients aged less than 50 years, follicle stimulating hormone (FSH) and luteinising hormone (LH) levels had to be within the post-menopausal range. Patients who had undergone bilateral surgical oophorectomy were also considered. The patients had to have received a previous systemic anti-cancer therapy which had been stopped at least 3 weeks before their entry into the study. The wash-out period was extended up to 6 weeks if drugs in depot formulation had been previously used.

Correspondence: E Bajetta, Division of Medical Oncology B, Istituto Nazionale per lo Studio e la Cura dei Tumori, Via Venezian 1, 20133 Milan, Italy
Received 5 January 1995; revised 27 April 1995; accepted 24 May 1995

Patients were excluded if they had endocrine disorders or other concurrent malignant disease or if they were taking concomitant anti-cancer treatments (with the exception of limited radiotherapy fields in the presence of other evaluable lesions). Patients were also considered ineligible if they had significant renal or hepatic dysfunction (creatinine > 1.25 times the upper limit of normal, bilirubin > 1.25 times the upper limit of normal and/or transaminases > 1.25 times the upper limit of normal). Patients with WBC and platelet counts of respectively $< 3000\ mm^{-3}$ and $< 100\,000\ mm^{-3}$ were excluded, as were those who showed more than one-third liver involvement, lymphangitic lung metastases or brain deposits. Staging and tumour response were defined by means of physical examination, bone scan, chest and skeletal radiographs, liver echography or computed tomographic scan, whole blood cell counts and blood chemistry. These examinations were performed at the beginning of the study, after 56 days as first evaluation, and then every 2 months. Signs, symptoms and toxicity were evaluated according to National Cancer Institute (Bethesda) criteria, and clinical response according to UICC criteria (Hayward *et al.*, 1977). All of the patients gave their informed consent and the study was approved by the local bioethics committee.

*Study design and treatment plan*

The study considered four consecutive treatment groups of 14 patients each. Exemestane was supplied by Pharmacia (Farmitalia Carlo Erba) in 2.5, 5, 12.5 and 25 mg capsules, and was taken p.o. at 12 a.m. daily. The first 14 patients entering the study received the highest dose of 25 mg $day^{-1}$; the next two groups of 14 patients respectively received 12.5 and 5 mg $day^{-1}$; the final 14 patients received 2.5 mg $day^{-1}$. The patients were seen in an out-patient setting on the day before treatment was started, when they were examined and staged. On the first day of treatment, blood samples were taken at 9 a.m. for endocrine studies and an overnight 12 h urine sample was also collected. The patients were subsequently examined on days 7, 14, 28 and 56, when toxicity was assessed and further blood samples were taken for haematological, biochemical and endocrine studies; urine samples were also collected on days 14, 28 and 56. Tumour response was also evaluated on day 56. Blood was collected at room temperature, allowed to clot, centrifuged at 3000 *g* and then stored at −20°C until assay. Oestrogen, LH, FSH, sex-hormone binding globulin (SHBG) and dehydroepiandrosterone sulphate (DHEAS) serum levels were all measured when the accrual for each dose group had been completed. In addition, while the study was ongoing and owing to the increasing importance given to $E_1S$ in the endocrinological evaluation of women with breast cancer in the recent literature, we decided to test $E_1S$ in order to complete the profile of drug activity. Unfortunately, for technical reasons, $E_1S$ levels could only be assessed in the last group of patients.

The patients were instructed how to collect overnight 12 h urine and each of them was given a standard plastic tube (volume 1 L, Kartell, Milan, Italy); in order to check that the patients collected all urine volume, they were asked to complete a personal card indicating when and how many times urine had been collected, especially during the night. On examination days, the patients had to return the tube and the card, which was checked by nursing staff. Thereafter, the volume of urine was measured and a 20 ml sample was taken and kept frozen at −20°C until analysis for 17-hydroxycorticosteroids (17-OHCS). If their disease had not progressed after 56 days of treatment, the patients continued receiving the same dose of exemestane; treatment was stopped at the time of documented disease progression.

*Hormonal measurements*

All hormone assays were performed by the Laboratory of Endocrinology at Milan's Istituto Nazionale per lo Studio e la Cura dei Tumori.

Serum $E_1$ and $E_2$ levels were measured by means of radio immunoassay (RIA) after liquid phase extraction and chromatographic separation. $^3H$-$E_1$ (Bio-Merieux) and $^3H$-$E_2$ (Bio-Merieux) were added to each serum sample (3 ml) as recovery markers and extracted with 11 ml diethyl ether. The serum phase was frozen and the ether extract decanted into clean tubes and dried under nitrogen. This free steroid-containing fraction was reconstituted in 1 ml of isooctane saturated with ethyleneglycol before proceeding to the chromatographic separation of $E_1$ and $E_2$ on a Celite column [Celite Supelco mixed with ethyleneglycol (2:1, w/v)]. In the chromatographic step, the redissolved sample was applied to the column and successive elutions with increasing concentrations of ethylacetate in isooctane (0, 18 and 40%) were collected. The fractions containing $E_1$ (18% ethylacetate) and $E_2$ (40% ethylacetate) were evaporated under nitrogen and the dried samples redissolved in 500 µl of the appropriate incubation buffer. A duplicate aliquot of this suspension (100 µl for $E_1$ and 50 µl for $E_2$) was subjected to the specific RIA procedure, and a further aliquot (200 µl for $E_1$ and for $E_2$) to final recovery, which ranged between 75% and 85% for $E_1$ and between 70% and 80% for $E_2$. The blank determined in the bidistilled water sample prepared in the same way as the serum sample did not exceed 2 pg per tube for $E_1$ and 0.25 pg per tube for $E_2$. The commercially available RIA kits $^3H$-$E_1$ (Bio Merieux) and $^{125}I$-$E_2$ (Clinical Assay) were used to determine $E_1$ and $E_2$ levels. The standard curve of the $E_2$ kit (supplied in serum) was substituted with a standard curve in buffer. The sensitivity of the assay was 1 pg $ml^{-1}$ for $E_2$ and 4 pg $ml^{-1}$ for $E_1$. The intra-assay coefficients of variation (CVs) ($n = 9$) for $E_2$ were 3.1% and 1.8% at 25 pg $ml^{-1}$ and 14 pg $ml^{-1}$ respectively; the intra-assay CVs for $E_1$ were 8.1% and 9.0% at 39 pg $ml^{-1}$ and 21 pg $ml^{-1}$ respectively. The interassay CV was 7.7% for $E_1$ and 6% for $E_2$ ($n = 11$).

Serum $E_1S$ levels were measured by means of RIA after enzyme hydrolysis, liquid phase extraction and chromatographic separation on a Celite column. A serum sample of 3 ml was extracted with 11 ml of diethyl ether to eliminate free steroids, and the ether phase discarded. $^3H$-$E_1S$ (Du Pont Nen Net-203 Estrone Sulphate, ammonium salt [6,7-$^3H(N)$]) was added to the aqueous phase (containing $E_1S$) as a recovery marker, and the solution was submitted to enzyme hydrolysis with 2 mg of sulphatase (Sigma 9626 type H-I) and 3 ml 0.2 M acetate buffer (pH 4.8) and then incubated for 22 h at 45°C. Extraction, chromatographic separation and RIA were carried out as for $E_1$. The final recovery was 70–80%. The blank determined in the bidistilled water sample prepared in the same way as the serum sample did not exceed 4 pg per tube. The sensitivity of the assay was 8.5 pg $ml^{-1}$; the intra-assay CV was: 6.1% at 191 pg $ml^{-1}$ ($n = 9$) and 5.7% at 425 pg $ml^{-1}$ ($n = 7$), and the interassay CV was 11%.

Immunoreactive assays were used to determine serum DHEAS, LH, FSH and SHBG levels.

The performance data for these assays were as follows: DHEAS (Sclavo Technogenetics), sensitivity 50 ng $ml^{-1}$, intra-assay CV 6.3% and interassay CV 7.1%; LH (Ares Serono), sensitivity 0.5 mUI $ml^{-1}$, intra-assay CV 1.1% and interassay CV 2.9%; FSH (Ares Serono), sensitivity 0.5 mUI $ml^{-1}$, intra-assay CV 1.6% and interassay CV 1.7%; SHBG (Orion Corporation), sensitivity 6.25 nmol $l^{-1}$, intra-assay CV 3.5% and interassay CV 7.1%. All of the samples from individual patients were analysed in the same run of the assay procedure and all of the assays were carried out in duplicate. Urinary adrenal glucocorticoid metabolite levels (µmol 12 $h^{-1}$) were determined by means of gas chromatography according to the method of Murphy and West (1966).

**Statistical methods**

Except for 17-OHCS, the original values were log-transformed before performing the analyses.

Descriptive statistics (means and 95% confidence intervals) were computed for all hormone levels by dose levels and times of assessment.

Repeated-measurement analyses of variance (ANOVA) were performed to assess the effect of log(dose), time and time × log(dose) interaction factors on hormone levels. The $F$-tests on within-subject effects were adjusted as described by Huynh and Feldt (1976). The adopted significance level was 5%.

The best overall response calculated from the time of its onset to the time of progression was considered for the duration of response. Time to treatment failure (TTF) was defined as the period from the date of starting treatment to the date of progression, and analysed using the Kaplan–Meier method.

### Results

### Patient characteristics

Between January 1992 and February 1993, 56 patients divided into four groups of 14 patients each were sequentially treated with exemestane. All of the patients were assessable in terms of their endocrinological profile and clinical response; their main characteristics are shown in Table I. The four groups were homogeneous in terms of age, ER status and the sites and extent of disease. PgR status was positive in 30, negative in seven and unknown in 19 patients. Approximately 54% of the patients were both ER and PgR positive. The DFI was long in the majority of patients, being less than 2 years in 13; only one patient (in the 12.5 mg group) had no DFI. In 27 patients, spontaneous menopause had lasted for more than 5 years. All of the patients had previously received tamoxifen (53 patients for metastatic disease, 12 patients also as adjuvant treatment). Twenty-three patients had previously received chemotherapy for advanced disease, six of whom had been treated with two or more regimens.

**Table I** Main patient characteristics

| | *25 mg* | *12.5 mg* | *5 mg* | *2.5 mg* |
|---|---|---|---|---|
| No. of patients | 14 | 14 | 14 | 14 |
| Median age | 61 | 60 | 63 | 56 |
| (range) | (40–75) | (37–78) | (49–75) | (34–68) |
| $ER^+$ | 10 | 11 | 11 | 11 |
| ER (fmol $mg^{-1}$): | | | | |
| 10–50 | 5 | 3 | 4 | 5 |
| >50 | 4 | 7 | 5 | 4 |
| Unknown | 4 | 3 | 3 | 3 |
| $ER^+/PgR^+$ | 6 | 9 | 9 | 6 |
| Spontaneous menopause | 9 | 7 | 9 | 7 |
| Oophorectomy | 2 | 5 | 4 | 4 |
| Drug-induced menopause | 3 | 2 | 1 | 3 |
| Site of metastatic disease: | | | | |
| Soft tissue | 6 | 3 | 7 | 6 |
| Viscera | 9 | 10 | 7 | 7 |
| Bone | 11 | 9 | 5 | 9 |
| Number of sites: | | | | |
| 1 | 5 | 7 | 9 | 6 |
| ⩾2 | 9 | 7 | 5 | 8 |
| Previous endocrine therapies for metastatic disease: | | | | |
| 1 treatment | 10 | 8 | 10 | 11 |
| >1 treatment | 4 | 4 | 3 | 3 |

### Endocrine effects

The serum values of $E_1$ and $E_2$ before and during treatment with each exemestane dose are reported in Table II. Maximum suppression was reached on day 7 and maintained thereafter. Figures 1 and 2 show the changes in $E_1$ and $E_2$ expressed as relative changes *vs* baseline values. There was also a progressive decrease in SHBG levels over time (Table III). Serum DHEAS (Table III) and urinary 17-OHCS (Table IV) levels remained unchanged during treatment at all of the tested doses. A tendency to increase over time was observed for gonadotrophin levels (Figure 3). The trend of $E_1S$ serum levels in the 2.5 mg group was in line with those of $E_1$ and $E_2$ (Table V, Figure 4).

**Figure 1** Relative changes *vs* baseline in serum $E_1$ levels during treatment for each exemestane dose in all treated patients.

**Figure 2** Relative changes *vs* baseline in serum $E_2$ levels during treatment for each exemestane dose in all treated patients.

**Table II** Serum $E_1$ and $E_2$ levels (pmol $l^{-1}$) during exemestane therapy, expressed as means

| $E_1$ | *25 mg day$^{-1}$* | *12.5 mg day$^{-1}$* | *5 mg day$^{-1}$* | *2.5 mg day$^{-1}$* |
|---|---|---|---|---|
| Day 0 | 71.0 (57.2–88.1)[a] | 91.9 (72.5–116.7) | 98.4 (78.1–123.9) | 75.2 (64.9–87.2) |
| 7 | 28.4 (24.1–33.4) | 29.7 (25.1–35.1) | 30.8 (25.2–37.7) | 30.1 (25.3–35.9) |
| 14 | 34.4 (29.4–40.2) | 30.9 (25.9–36.8) | 30.6 (24.7–37.8) | 33.1 (28.4–38.5) |
| 28 | 37.6 (32.7–43.1) | 32.4 (25.1–41.8) | 32.1 (26.9–38.2) | 35.4 (30.2–41.5) |
| 56 | 35.5 (30.2–41.7) | 35.7 (29.7–42.9) | 35.9 (29.8–43.4) | 35.8 (30.2–42.3) |
| $E_2$ | | | | |
| Day 0 | 15.3 (12.6–18.5) | 18.9 (14.6–24.4) | 20.6 (14.9–28.6) | 12.6 (10.9–14.6) |
| 7 | 5.2 (4.8–5.5) | 5.8 (5.0–6.7) | 6.2 (5.3–7.2) | 5.9 (5.4–6.6) |
| 14 | 5.3 (5.1–5.9) | 5.8 (5.0–6.7) | 5.7 (4.9–6.5) | 6.0 (5.3–6.8) |
| 28 | 5.5 (5.1–6.0) | 5.9 (5.1–6.9) | 6.3 (5.4–7.4) | 6.0 (5.4–6.5) |
| 56 | 5.3 (4.9–5.8) | 6.3 (5.4–7.4) | 6.4 (5.4–7.6) | 6.3 (5.6–7.0) |

[a]95% confidence interval in parentheses.

**Table III** Serum SHBG (nmol $l^{-1}$) and DHEA-S (ng $ml^{-1}$) during exemestane therapy, expressed as means

| *SHBG* | *25 mg day⁻¹* | *12.5 mg day⁻¹* | *5 mg day⁻¹* | *2.5 mg day⁻¹* |
|---|---|---|---|---|
| Day 0 | 60.7 (49.7–74.1)[a] | 64.0 (52.5–78.1) | 78.1 (53.4–114.0) | 54.1 (38.5–76.1) |
| 7 | 52.0 (42.2–64.1) | 60.2 (49.9–72.6) | 81.0 (54.7–120.1) | 52.9 (37.9–73.9) |
| 14 | 48.5 (38.3–61.5) | 53.4 (43.5–65.5) | 76.9 (50.2–117.8) | 47.9 (34.6–66.3) |
| 28 | 41.9 (30.8–57.0) | 50.9 (42.1–61.6) | 67.2 (43.2–104.4) | 44.6 (32.8–60.5) |
| 56 | 35.3 (25.3–49.3) | 47.5 (37.6–59.9) | 61.8 (41.7–91.6) | 40.9 (30.2–55.6) |
| *DHEA-S* | | | | |
| Day 0 | 868.5 (610.9–1234.6) | 886.0 (655.5–1197.6) | 979.0 (721.8–1327.9) | 1076.1 (811.6–1426.8) |
| 7 | 960.5 (678.5–1359.6) | 710.4 (501.7–1006.1) | 878.6 (637.1–1211.6) | 1006.1 (756.7–1337.6) |
| 14 | 963.6 (676.9–1371.6) | 725.9 (502.4–1048.9) | 803.9 (611.1–1057.7) | 1060.5 (806.2–1394.9) |
| 28 | 984.6 (657.9–1473.5) | 736.8 (521.4–1041.3) | 791.4 (544.5–1150.4) | 1088.2 (835.1–1418.0) |
| 56 | 1033.6 (680.8–1569.2) | 685.5 (471.3–996.9) | 807.2 (578.7–1125.9) | 1060.9 (815.8–1379.6) |

[a]95% confidence interval in parentheses.

**Table IV** Urinary 17-OHCS (µmol 12 $h^{-1}$) during exemestane therapy, expressed as means

| *17-OHCS* | *25 mg day⁻¹* | *12.5 mg day⁻¹* | *5 mg day⁻¹* | *2.5 mg day⁻¹* |
|---|---|---|---|---|
| Day 0 | 5.1 (4.3–6.0)[a] | 6.8 (4.9–8.6) | 5.8 (3.7–7.9) | 6.3 (4.8–7.9) |
| 14 | 5.7 (4.3–7.1) | 6.8 (5.4–8.2) | 5.7 (3.8–7.7) | 5.4 (4.4–6.5) |
| 28 | 5.2 (4.3–6.0) | 5.3 (4.2–6.4) | 4.9 (3.5–6.3) | 5.7 (4.2–7.3) |
| 56 | 5.1 (4.0–6.2) | 5.9 (4.4–7.5) | 5.5 (4.0–6.9) | 4.9 (3.8–6.0) |

[a]95% confidence interval in parentheses.

**Table V** Serum $E_1S$ (pmol $l^{-1}$) during exemestane therapy, expressed as means (assay performed only in 2.5 mg group)

| *E₁S* | *2.5 mg day⁻¹* |
|---|---|
| Day 0 | 606.2 (475.4–773.0)[a] |
| 7 | 158.9 (124.6–202.7) |
| 14 | 164.0 (131.1–205.2) |
| 28 | 149.9 (125.6–179.0) |
| 56 | 189.6 (148.3–242.6) |

[a]95% confidence interval in parentheses.

**Figure 3** Changes in serum LH and FSH (mIU $ml^{-1}$) levels during treatment for each exemestane dose, expressed as the mean for all treated patients.

Between-group comparisons of baseline hormone values showed no difference for any hormone except $E_2$ ($P = 0.024$), with the $E_2$ baseline values being lower in the 2.5 mg group. At ANOVA, a significant interaction time × log(dose) ($P = 0.0476$) was found only for $E_2$, the degree of suppression appearing to be less for the lower dose. A significant time factor effect was found for the suppression of $E_1$ and $E_2$ ($P < 0.0001$). $E_1S$ in the 2.5 mg dose group was also

**Figure 4** Relative changes *vs* baseline in serum $E_1S$ levels during treatment in the 2.5 mg group as a whole.

decreased ($P < 0.0001$), whereas there was a significant increase in LH ($P = 0.0375$) and FSH ($P = 0.0046$). The levels of none of the other hormones significantly changed during treatment.

*Clinical toxicity*

The tolerability of each exemestane dose was highly satisfactory. There were no serious side-effects which could be attributed to the drug, and no patient had to discontinue treatment. Most of the symptoms were mild (grade 1 NCI) and transient. The main side-effects involved the gastrointestinal system: nausea, abdominal pain and diarrhoea in respectively 12.5%, 7% and 3.6% of the patients. Hot flushes, an allergic reaction and dizziness were reported by one patient each; two patients experienced headache. No haematological or biochemical toxicity was observed at any dose level. In one patient treated with exemestane 12.5 mg, atrial arrhythmia (grade 2 NCI) occurred.

*Control of tumour growth*

All of the 56 patients were evaluable and, irrespective of exemestane dose, there was an overall response rate of 18% (10/56). Two patients on exemestane 5 mg achieved a complete response lasting 11 and >20 months respectively. They both had positive receptors, spontaneous menopause lasting more than 5 years and only skin disease. Partial responses were achieved at the other three doses (three at 25 mg, two at 12.5 mg and three at 2.5 mg), with a median duration of 13 months (range 2–18+). Soft tissue lesions represented the most responsive sites (14/32); only two patients with bone disease responded, and one patient affected by liver metastasis achieved a partial response lasting 14 months. Stable disease with a median duration of 6 months (range 4–27) was obtained in 27 cases; the remaining 19 patients experienced disease progression. The TTF is given in Figure 5.

**Figure 5** Time to treatment failure for all entered patients; *n* = number of patients.

### Discussion

Exemestane is a novel orally active irreversible aromatase inhibitor and our study shows its effectiveness in suppressing both serum $E_1$ and $E_2$ levels when given in repeated doses. A significant reduction in serum $E_1S$ oestrogen levels was also obtained with the lowest dose of 2.5 mg. The difference in the percentage of $E_2$ reduction obtained with the lowest dose does not seem to be due to any lesser efficacy in inhibiting $E_2$ synthesis, but more probably to the lower $E_2$ baseline values of these patients. Our data also show an effective reduction of $E_1S$ levels in the 2.5 mg group, similar to that observed by Evans *et al.* (1992) using higher single doses.

A statistically significant increase in serum LH and FSH levels was observed during treatment, a finding which has already been reported in other studies using orally administered aromatase inhibitors (Dowsett and Coombes, 1994). This increase is probably the result of a feedback mechanism due to the reduction in circulating oestrogen levels that stimulates the pituitary secretion of gonadotrophins also in post-menopausal women.

It is known that SHBG reflects the oestrogen–androgen balance as its synthesis is stimulated by oestrogens and inhibited by androgens. In post-menopausal women, SHBG is mainly a marker of androgenic activity. In our study, we found some decrease in SHBG (although this was not statistically significant) and so our data do not support the androgenic activity of the drug. A fall in SHBG has also been shown following high oral doses of formestane (Dowsett and Coombes, 1994), and has been explained by the rapid increase in drug levels in the liver (the site of SHBG synthesis) following oral absorption. This has not been previously reported for the parenteral administration, of even high doses, probably owing to the short duration of the hormonal study (Goss *et al.*, 1986); in a previous experience, we observed a significant decrease in SHBG in patients receiving formestane i.m. but only from the second month of treatment onward (Zilembo *et al.*, 1994). The effects of all of the exemestane doses persisted over time and appeared to be greater than those we have previously described for the other steroidal aromatase inhibitor formestane (Bajetta *et al.*, 1994) which, in comparison with baseline, led to an average decrease in $E_2$ serum levels of 40% after 15 days that remained subsequently unchanged, with no difference between the two doses of 250 and 500 mg i.m. every 2 weeks. The efficacy of exemestane in reducing oestrogen levels appears to be lower than that of synthetic derivatives such as letrozole and vorozole, which are capable of reducing oestrogen levels to undetectable levels. In a phase I study with letrozole, Iveson *et al.* (1993) found that $E_1$ and $E_2$ plasma levels were suppressed to below the detection limit of the assays regardless of the doses (0.1, 0.5 and 2.5 mg p.o. $day^{-1}$). The same result was obtained using racemic vorozole at once daily doses of 2.5 or 5 mg (Borms *et al.*, 1991). In both treatment arms, plasma $E_2$ levels dropped to below the detection limit of the assay within 1 month of treatment. Although the reduction in circulating oestrogen levels is considered to be an expression of the drugs' effectiveness, no clinical relationship has been found (i.e. the higher the oestrogen reduction, the greater the clinical response), and so it is likely that these drugs have more than one mechanism of action. Our data confirm the selectivity of exemestane because no effect on adrenal steroidogenesis was documented by the 17-OHCS urinary excretion measurements.

In our experience, exemestane is a well-tolerated endocrine treatment for advanced breast cancer patients; even the gastrointestinal symptoms which are generally more frequent with oral formulations were reported by only 16% of the patients, and were transient, very mild and not dose related.

In terms of clinical efficacy, our response rate appears to be quite low but, in our opinion, this may be due to the large number of previous treatments. In any case, it was particularly encouraging that two complete responses were obtained, and that three partial responses were obtained with the lowest dose; furthermore, a good number of patients (27%) achieved disease stabilisation lasting at least 6 months.

In conclusion, we think that exemestane may be a useful option in the management of endocrine-dependent advanced breast cancer, and that the good tolerability of the orally administered drug may increase the number of patients suitable for treatment with aromatase inhibitors.

**Acknowledgements**
The authors wish to thank the team of data managers from the Italian Trials in Medical Oncology Group (ITMO) for their collaboration.

### References

BAJETTA E, ZILEMBO N, BUZZONI R, NOBERASCO C, CELIO L AND BICHISAO E. (1993). Efficacy and tolerability of 4-hydroxyandrostenedione (4-OHA) as first-line treatment in postmenopausal patients with breast cancer after adjuvant therapy. *Cancer Treat. Rev.*, **19**, 31–36.

BAJETTA E, ZILEMBO N, BUZZONI R, NOBERASCO C, DI LEO A, BARTOLI C, MERSON M, MOGLIA D, CELIO L AND NELLI P. (1994). Endocrinological and clinical evaluation of two doses of formestane on advanced breast cancer. *Br. J. Cancer*, **70**, 145–150.

BORMS M, BRUYNSEELS J, DE COSTER R AND JANSSEN PAJ. (1991). R76713: non-steroidal aromatase inhibitor. Phase I open pilot trial in the endocrine treatment of advanced post-menopausal breast cancer. *Proc. 4th Int. Congress on Hormones and Cancer, Amsterdam, September 15–19 1991*, p. 237.

COOMBES RC, GOSS PE, DOWSETT M, GAZET JC AND BRODIE AMH. (1984). 4-Hydroxyandrostenedione in the treatment of postmenopausal patients with advanced breast cancer. *Lancet*, **2**, 1237–1239.

COOMBES RC, HUGHES SWM AND DOWSETT M. (1992). 4-Hydroxyandrostenedione: a new treatment for postmenopausal patients with breast cancer. *Eur. J. Cancer*, **28**, 1941–1945.

DI SALLE E, GUIDICI D, BRIATICO G AND ORNATI G. (1990). Novel irreversible aromatase inhibitor. *Ann. N.Y. Acad. Sci.*, **595**, 357–367.

DI SALLE E, ORNATI G, GIUDICI D, LASSUS M, EVANS TRJ AND COOMBES RC. (1992). Exemestane (FCE 24304), a new steroidal aromatase inhibitor. *J. Steroid Biochem. Mol. Biol.*, **43**, 137–143.

DOWSETT M AND COOMBES RC. (1994). Second generation aromatase inhibitor, 4-hydroxyandrostenedione. *Breast Cancer Res. Treat.*, **30**, 81–87.

DOWSETT M, CUNNINGHAM DC, STEIN RC, EVANS S, DEHENNIN L, HEDLEY L AND COOMBES RC. (1989). Dose-related endocrine effects and pharmacokinetics of oral and intramuscular 4-hydroxyandrostenedione in postmenopausal breast cancer patients. *Cancer Res.*, **49**, 1306–1312.

EVANS TRJ, DI SALLE E, ORNATI G, LASSUS M, STROLIN BENEDETTI M, PIANEZZOLA E AND COOMBES RC. (1992). Phase I and endocrine study of exemestane (FCE 24304), a new aromatase inhibitor, in postmenopausal women. *Cancer Res.*, **52**, 5933–5939.

FERRARI L, ZILEMBO N, BAJETTA E, BUZZONI R, NOBERASCO C, MARTINETTI A, CELIO L, GALANTE E, OREFICE S AND CERROTTA AM. (1994). Effect of two 4-hydroxyandrostenedione doses on serum insulin-like growth factor I levels in advanced breast cancer. *Breast Cancer Res. Treat.*, **30**, 127–132.

GIUDICI D, ORNATI G, BRIATICO G, BUZZETTI F, LOMBARDI P AND DI SALLE E. (1988). 6-Methylenandrosta-1,4-diene-3,17-dione (FCE 24304): a new irreversible aromatase inhibitor. *J. Steroid Biochem.*, **30**, 391–394.

GOSS PE, POWELS TJ, DOWSETT M, HUTCHISON G, BRODIE AMH, GAZET JC AND COOMBES RC. (1986). Treatment of advanced postmenopausal breast cancer with an aromatase inhibitor, 4-hydroxyandrostenedione: Phase II report. *Cancer Res.*, **46**, 4823–4826.

HAYWARD JL, CARBONE PP, HEUSON JC, KUMOOKA S, SEGALOFF A AND RUBENS RD. (1977). Assessment of response to therapy in advanced breast cancer. *Eur. J. Cancer*, **13**, 89–94.

HÖFFKEN K, YONAT W, POSSINGER K, KOLBEL M, WAGNER TKH, BECHER R, COLLIES R, FRIEDERICH P, WILLMANNS W, MAASS H AND SCHMIDT CG. (1990). Aromatase inhibition with 4-hydroxyandrostenedione in the treatment of postmenopausal patients with advanced breast cancer: a Phase II study. *J. Clin. Oncol.*, **8**, 875–880.

HUYNH H AND FELDT LS. (1976). Conditions under which mean square ratios in repeated measurement designs have exact F-distribution. *J. Am. Stat. Ass.*, **65**, 1582–1589.

IVESON TJ, SMITH JE, AHERN J, SMITHERS DA, TRUNET PF AND DOWSETT M. (1993). Phase I study of the oral nonsteroidal aromatase inhibitor CGS20267 in postmenopausal patients with advanced breast cancer. *Cancer Res.*, **53**, 266–270.

MURPHY D AND WEST HF. (1966). Urinary 17-hydroxycorticosteroids measured by gas-chromatography. *J. Endocrinol.*, **36**, 331–340.

PICKLES T, PERRY L, MURRAY P AND PLOWMAN P. (1990). 4-Hydroxyandrostenedione – further clinical and extended endocrine observations. *Br. J. Cancer*, **62**, 309–313.

REED MJ, CHRISTODOULIDES A, KOISTINEN R, SEPPALA M, TEALE JD AND GHILCHIK MW. (1992). The effect of endocrine therapy with medroxyprogesterone acetate, 4-hydroxyandrostenedione or Tamoxifen on plasma concentrations of insulin-like growth factor (IGF)-I, IGF-II and IGFBP-1 in women with advanced breast cancer. *Int. J. Cancer*, **52**, 208–212.

STEIN RC, DOWSETT M, HEDLEY A, DAVENPORT J AND COOMBES RC. (1990). Treatment of advanced breast cancer in postmenopausal women with 4-hydroxyandrostenedione. *Cancer Chemother. Pharmacol.*, **26**, 75–78.

ZACCHEO T AND DI SALLE E. (1989). Effect of the irreversible aromatase inhibitor FCE 24304 on DMBA-induced mammary tumors in ovariectomized rats treated with testosterone. *Cancer Chemother. Pharmacol.*, **25**, 95–98.

ZACCHEO T, GIUDICI D, LOMBARDI P AND DI SALLE E. (1989). A new irreversible aromatase inhibitor, 6-methylenandrosta-1,4-diene-3,17-dione (FCE 24304): antitumor activity and endocrine effects in rats with DMBA-induced mammary tumors. *Cancer Chemother. Pharmacol.*, **23**, 47–50.

ZILEMBO N, BUZZONI R, CELIO L, NOBERASCO C, FERRARI L, LAFFRANCHI A, DI MAURO MG, DOLCI S AND BAJETTA E. (1994). Formestane as treatment of advanced breast cancer in elderly women. *Tumori*, **80**, 433–437.